# Glyphosate residue concentrations in honey attributed through geospatial analysis to proximity of large-scale agriculture and transfer off-site by bees

**DOI:** 10.1371/journal.pone.0198876

**Published:** 2018-07-11

**Authors:** Carl J. Berg, H. Peter King, Glenda Delenstarr, Ritikaa Kumar, Fernando Rubio, Tom Glaze

**Affiliations:** 1 Kauaʽi Chapter, Surfrider Foundation, Līhuʽe, Hawaii, United States of America; 2 DataWise Consulting, Kapaʽa, Hawaii, United States of America; 3 Delenstarr Consulting LLC, Kōloa, Hawaii, United States of America; 4 Southern Oregon University, Ashland, Oregon, United States of America; 5 Abraxis Inc., Warminster, Pennsylvania, United States of America; Montana State University Bozeman, UNITED STATES

## Abstract

Honey taken directly from 59 bee hives on the Hawaiian island of Kauaʽi was analyzed for glyphosate residue using ELISA techniques. Glyphosate residue was detected (> LOQ) in 27% of honey samples, at concentrations up to 342 ppb, with a mean = 118 ppb, S.E.M. 24 ppb. Of 15 honey samples store-purchased on Kauaʽi, glyphosate was detected in 33%, with a mean concentration of 41 ppb, S.E.M. 14. Glyphosate residue was not detected in two samples from the island of Molokai but was in one of four samples from the island of Hawaiʽi. Presence and concentration of glyphosate residues were geospatially mapped with respect to Hawaiian land divisions. Mapping showed higher occurrence of glyphosate that was over LOQ (48%) and concentrations of glyphosate (mean = 125 ppb, S.E.M. 25 ppb; N = 15) in honey from the western, predominantly agricultural, half of Kauaʽi versus the eastern half (4%, mean = 15 ppb; N = 1). Geographic Information System analysis of land use percentage was performed within a circular zone of 1 Km radius around each hive. Various land use types within each circular zone were transcribed into polygons and percent land use calculated. Only agriculture land use showed a strong positive correlation with glyphosate concentration. High glyphosate concentrations were also detected when extensive golf courses and/or highways were nearby. This suggests herbicide migration from the site of use into other areas by bees. Best management practices in use for curtailing pesticide migration are not effective and must be carefully re-assessed.

## Introduction

Rubio et al. [1] tested for glyphosate residues in honey and other common foodstuff and described an enzyme-linked immunosorbent assay (ELISA) technique for inexpensive analysis of the General Use Pesticide Glyphosate (N-(phosphonomethyl) glycine), sold as Monsanto’s Roundup™. This herbicide is widely used in commercial agriculture, on golf courses, roadsides, and around homes and gardens. They found glyphosate in 45.5% of honey samples that were labeled as Organic. They also found glyphosate in a greater percentage of their samples (70%) from countries that allowed genetically modified organisms (GMO) selected for glyphosate resistance than samples (21%) from those countries that did not. More recent studies have shown glyphosate residues in other food, including soy, cereals, and ice cream [2]. Tolerance levels have been established for residues of glyphosate in food, including its metabolites and degradates [3], but none have been established for honey. Contamination of processed food may occur, in a small part, if honey is an ingredient in the processed food. The U.S. Food and Drug Administration does not test for glyphosate for its annual Pesticide Residue Monitoring Program, thus the extent of food contamination by glyphosate in the United States is unknown. The Canadian Food Inspection Agency reported glyphosate in 29.7% of 3,188 food samples tested in 2015–2016 [4].

Glyphosate may pose a public health risk, leading to world-wide concern and social action, especially as it has already entered the human food chain [5]. Recent research has shown the prevalence rate and mean concentration of glyphosate in human urine increased between 1993 and 2016 [5, 6]. Possible mechanisms underlying glyphosate toxicity in mammals have recently been described [7–9]. The World Health Organization’s International Agency for Research on Cancer report for 2016 [2] summarizes the scientific data and, based on that report, the State of California listed glyphosate as known to cause cancer and products must be labeled as such [10].

The mechanisms whereby glyphosate moves from sites of application to foodstuffs is variable but includes direct application on the food crop, migration off-site via air drift or water flow, through contamination during harvesting and processing, or carrying by animals, *e*.*g*. bees. [11–14].

The Hawaiian island of Kauaʽi is called the Garden Island because of both its verdant landscape and its extensive agriculture. Once dominated by sugar and pineapple industries, Kauaʽi now hosts large-scale agriculture of coffee, corn grown as a seed crop, and large-scale experimental crop testing for plants’ resistance to pesticides, including glyphosate [15, 16]. Glyphosate is also used on Kauaʽi on roadsides, irrigation ditches, home gardens, landscaping, and on golf courses. Great citizen concern led to Kauaʽi County Bill 2491 and Ordinance 960 in 2013 for the formation of a Joint Fact Finding study on “Pesticide Use by Large Agribusinesses on Kauaʽi” [15]. The study brought together available information on pesticide usage by GMO seed corn and coffee crops, as well as any possible impacts to environmental and human health. Preliminary discovery of glyphosate in honey was presented in the report and led to the more extensive investigation reported here in both hive collected and market purchased samples.

Furthermore, application of spatial intelligence, the process of deriving meaningful insight from geospatial data relationships, was applied to the current study of glyphosate contamination in honey taken directly from the hives by correlating the location of the hive with respect to geographical features and land use. Two independent methods on two separate datasets (Google Earth Pro™ and NOAA C-Cap) were tested to determine the most efficient and accurate determination of land use concurrent with honey sampling. Geospatial identification of dominant land use near hives indicates a source and pathway for glyphosate to enter the food chain.

The main objective of this study was to determine glyphosate concentration in honey obtained on Kauaʽi and, using geospatial analysis, determine those land use attributes most correlated with glyphosate prevalence and concentration in the honey.

## Materials and methods

### Sample collection

Honey samples were collected directly from hives by beekeepers on the island of Kauaʽi in three batches from 2013 through 2016 (Table 1). Samples were opportunistically obtained from all accessible parts of the island. Collections were constrained by lack of bee hives in the area or beekeepers’ unwillingness to provide samples. A strict confidentiality agreement was needed to get participation in the study. For some sites, sample batches were collected over time, to increase sample size. The timing was irrespective of seasonality of honey production by the bees. Each sample came from a single unique hive and its location was precisely recorded. Two other batches of honey were obtained from merchants and comprised honey from many hives under control of the manufacturing company.

**Table 1:** Honey collection data and laboratory where glyphosate was analyzed by ELISA.

Batch Number	Sample ID	Date Collected	ELISA analysis location
**Hive Samples**			
Batch #1	37, 38	Fall 2013	Micro Inotech Lab.
Batch #2	1 to 36	Summer 2015	Surfrider Lab.
Batch #3	39 to 59	Fall 2016	Surfrider Lab.
**Merchant Samples**			
Batch #4	91 to 23	Winter 2016	Abraxis Lab.
Batch #5	60, 61, 62	Winter 2016	Surfrider Lab.

In the fall of 2013 (Batch 1) two honey samples were collected by beekeepers, by scraping the honey comb with the open mouth of a clean glass mason jars and sealing the jars. These samples were stored at room temperature in a closed box, in a cabinet, until shipment to Microbe Inotech Laboratories, Inc., St. Louis, MO, for analysis of glyphosate concentration.

During the spring of 2015 (Batch 2) 36 samples of honey were collected directly from their unique hives by beekeepers of Kauaʽi, using only the certified pre-cleaned 40 ml amber borosilicate glass vials provided to collect and store the honey. Vials were immediately sealed under a signed and dated custody seal by the collector and delivered directly to one of the authors (CJB, RK), along with a signed confidentiality statement containing contact information, date of collection, and hive location. Samples were stored at room temperature in a closed box, in a cabinet until shipment for analysis.

In fall of 2016 (Batch #3) 21 samples were collected by beekeepers and delivered to one of the authors (CJB), under the same procedures and stored for shipment as Batch #2.

In the winter of 2016 (Batch #4) 23 samples of honey were purchased from local famers’ markets, produce stands, and stores. Honey was decanted into glass vials, sealed, and stored as above. Commercially produced honey is a composite from many hives. Source location was broadly determined from the label or from discussion with merchants. Date of honey collection is unknown. Samples were sent to Abraxis Inc, laboratory for analysis.

Batch #5 comprises three honey samples. Two samples were from the island of Molokai. One was purchased at a store on Molokai and the other was obtained from the beekeeper’s bottled supplies. Both samples were a composite from hives at each beekeepers’ farm. The farms’ hives, which were located on Google Earth Pro™, were widely separated and thus represented different bee foraging sites. The third sample was purchased at a Kauai store and the source locality identified as from the island of Hawaii by its label.

### Sample analysis

ELISA analysis was performed at each laboratory using the Abraxis method [1]. Abraxis test kit (cat. # 500086) and microtiter equipment were used. The sample preparation method for honey followed published procedures [1, 17] (S1 Appendix). Samples were processed and read with a microplate reader Model 4303 [18] from Abraxis Inc. and analyzed using Molecular Devices Soft max pro evaluation program (4-Parameter). Results from Surfrider laboratory analysis were certified correct by Abraxis staff. Limit of quantitation (LOQ) was 15 ng/mL (15 ppb). Samples are stated as having detectable levels of glyphosate only if they are > LOQ.

Abraxis’ ELISA methods for analysis of glyphosate have been compared to standard liquid chromatography and tandem mass spectrometry methods but not for honey. Therefore, 14 samples from Batch #2 were analyzed by both methods for validation. The results closely correlated with R^2^ = 0.99 (S2 Appendix). Only ELISA derived data were used in this study.

### Geospatial analysis

**Fig 1:**
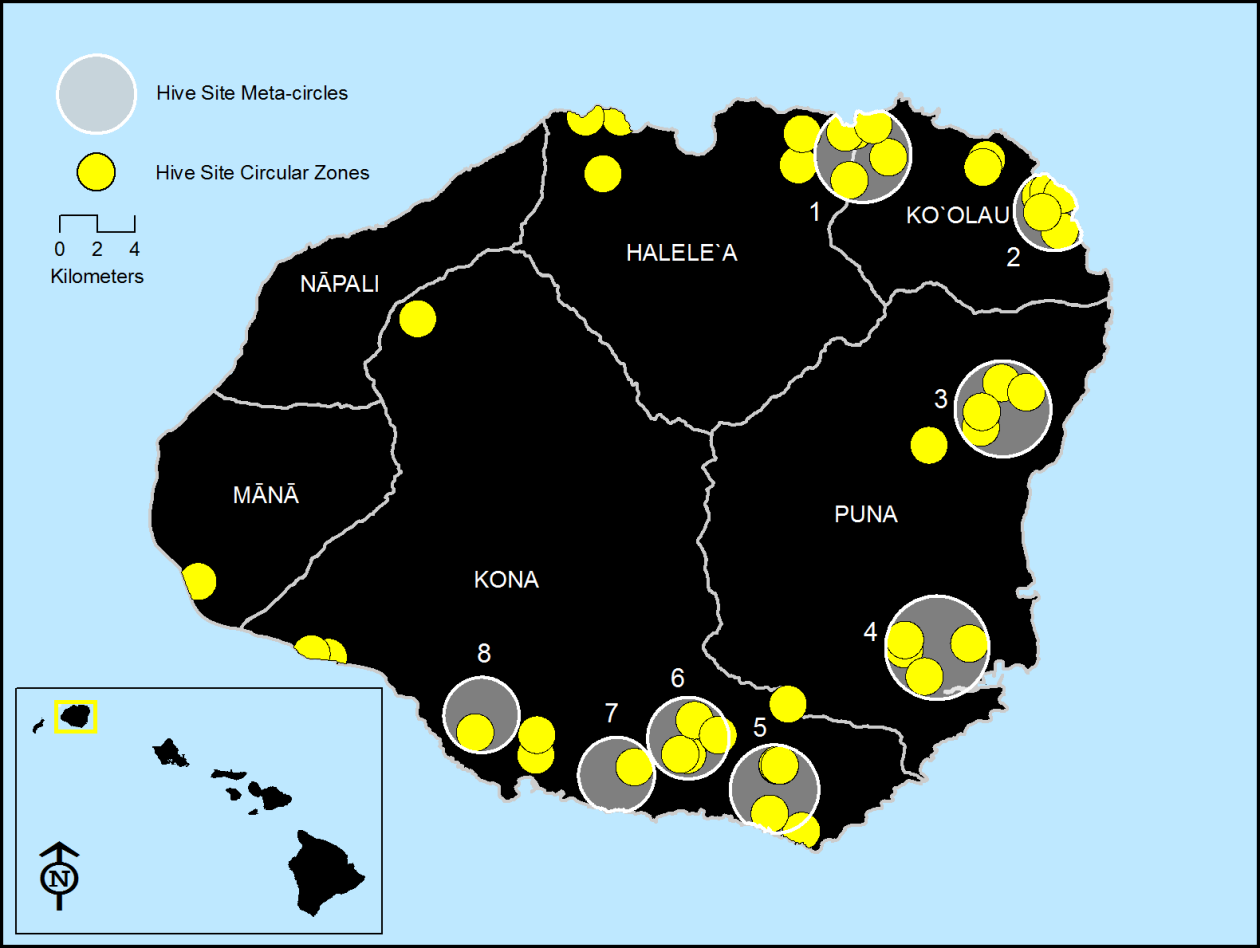
Distribution of 1 Km radius circular zones (yellow) around hives on island of Kauaʽi. Meta-circles of grouped circular zones are shaded in grey and numbered (N = 8). Moku divisions are indicated by white lines and each Moku is named.

Presence and concentration of glyphosate residues were geospatially mapped with respect to general geography of the island and land use. Ancient Hawaiian biogeographical and management land divisions (Moku) (Fig 1) [19] were identified using the Google Earth Pro™ (GEP).

### Circular zones

Bees have been reported to forage as far as 9.5 Km from the hive [20,21] with a mean distance closer to 1 Km at times subject to patchiness of flowering resources [21]. Depending upon resource availability, the probability of plant visitation decreased non-linearly from the hive and >85% probability of visitation was at less than 1 Km [22]. Beekeepers note that bees forage as close to the hive as possible [23], especially on Kauaʽi where naturally occurring plants and crops bloom year-round. Foraging on Kauai may also be constrained by discrete watersheds, bounded by mountainous ridges.

Based on this information, and to avoid overlapping of foraging sites, a 1 Km radius was used to define the bees’ foraging zone around each hive. Geospatial information analysis was applied using the GEP program with Digital Globe™ (DG) images to delineate circular zones 1 Km in radius around each hive (Fig 1).

The land area within each circular zone was further sub-divided into discrete polygons, based upon land cover designations derived from NOAA C-CAP twenty-one classifications [24] (Table 2). Habitat codes were reclassified to seven land use categories.

**Table 2:** Land use NOAA C-CAP classification descriptions.

This Study	Land use category	C-CAP	Land use classifications	Description of ground cover
		1	Unclassified	
1	Urban	2	High Developed	heavily built-up urban centers as well as large constructed surfaces in suburban and rural areas. Large buildings
		3	Medium Developed	constructed surface mixed with substantial amounts of vegetated surface. Small buildings
2	Suburban/Rural	4	Low Developed	class 3, with the addition of streets and roads with associated trees and grasses
3	Developed Open	5	Developed Open	parks, lawns, athletic fields, golf courses, and natural grasses occurring around airports and industrial sites
4	Agriculture	6	Orchard	herbaceous (cropland) and woody (e.g., orchards, nurseries, and vineyards) cultivated lands
		7	Pasture land	grasses, legumes or grass-legume mixtures planted for livestock grazing or the production of seed or hay crops
		8	Grassland	Grassland: grasses and non-grasses (forbs) that are not fertilized, cut, tilled, or planted regularly
		20	Bare land	bare soil, rock, sand, silt, gravel, or other earthen material with little or no vegetation
5	Forest	9	Deciduous forest	Deciduous Forest areas dominated by single stemmed, woody vegetation
		10	Evergreen forest	67 percent of the trees remain green throughout the year. Both coniferous and broad-leaved
		11	Mixed Forest	areas in which both evergreen and deciduous trees are growing and neither predominate
		12	Scrub/shrubs	woody vegetation: true shrubs,young trees, and trees or shrubs that are small
6	Wetland/Riparian	13	Palustrine Forested Wetland	non-tidal wetlands dominated by woody vegetation >5m
		14	Palustrine Scrub/Shrub Wetland	non-tidal wetlands dominated by woody vegetation less than or equal to 5 meters
		15	Palustrine Emergent Wetland	non-tidal wetlands dominated by persistent emergents, emergent mosses, or lichens
		16	Estuarine Forest Wetland	tidal wetlands dominated by woody vegetation >5m, salinity >0.5ppt
		17	Estuarine Scrub/Shrub Wetland	tidal wetlands dominated by woody vegetation <5m, salinity >0.5ppt
		18	Estuarine Emergent Wetland	erect, rooted, herbaceous hydrophytes. Perennial plants usually dominate these wetlands
7	Water	19	Unconsolidated Shore	substrates lacking vegetation: beaches, bars, and flats
		21	Open water	open water with less than 25 percent cover of vegetation or soil.

**Fig 2:**
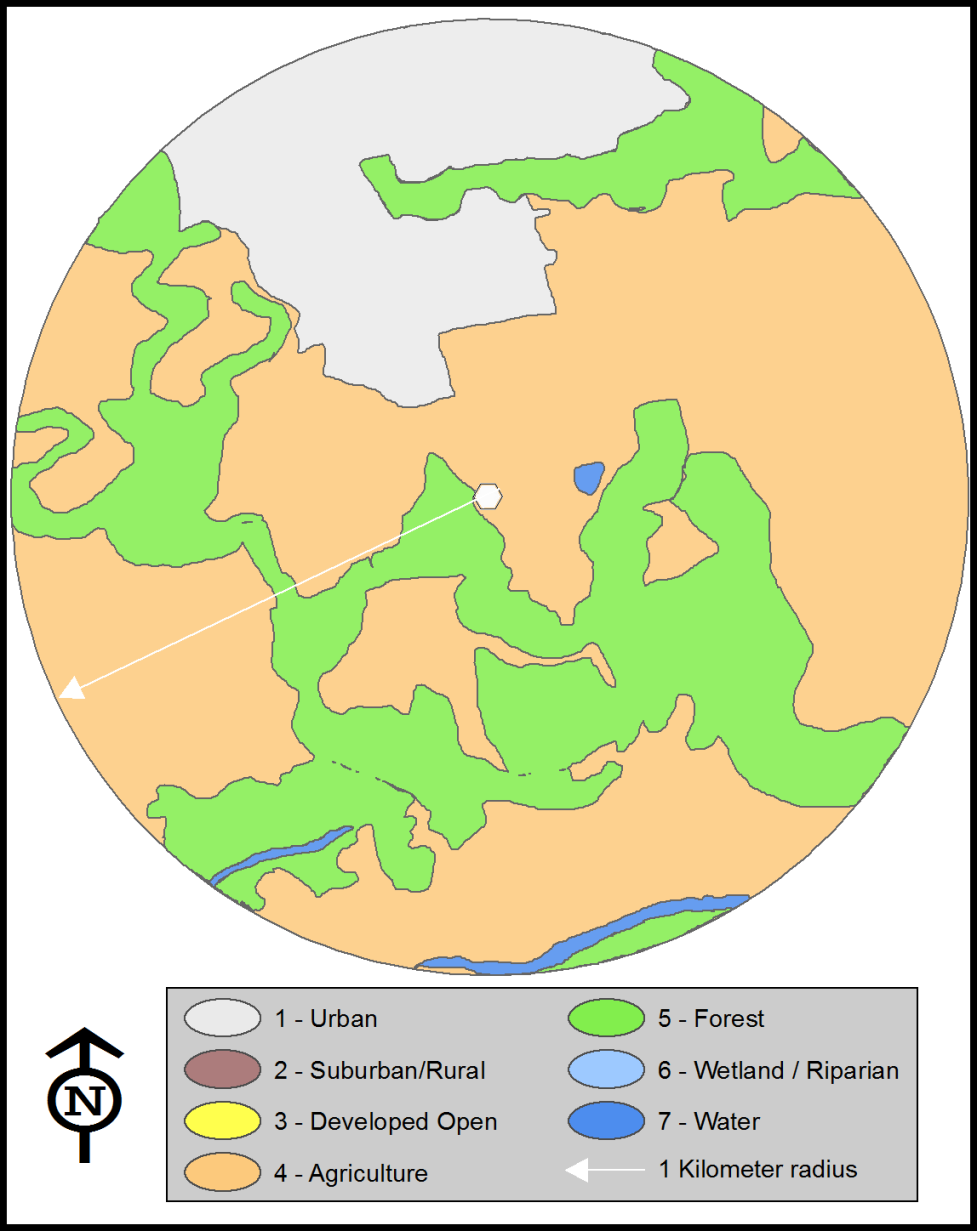
Circular zone around a central hive, drawn with 1 Km radius. Polygons represent different land uses categories. Site #16 provided as an illustration.

Individual polygons were delineated in GEP using an optical mouse and area covered was calculated. The land area of each habitat type was then summed to provide a measure of the total land area (m^2^) in each land use polygon (Fig 2). Each circular zone comprised 314.16 hectares, unless ocean area was excluded. A total of 18,872 hectares of land area were processed using the latest GEP images (years 2013–2014) and knowledge of current land use. Visual ground truthing was performed on sites known to differ from GEP images.

The percent of the current land use was calculated for each habitat type represented by the polygons within the hive sites’ circular zones. These percentages were then correlated with the concentration of glyphosate residue from the hive in the circular zone. One hive (#48, Mānā Moku) was excluded from polygon land use calculations, as it had been moved among sites within the Moku.

A second independent geospatial analytical method for land use categorization used the NOAA Coastal–Change Analysis Program (C-CAP) [24] and ArcGIS Version 10.5 [25] (S3 Appendix). It derived area (m^2^) within the 1 Km radius circular zones using a program that automatically identified different types of ground cover (Table 2). A comparison of the two methods for accuracy in determining current land use patterns showed GEP preferable, so it was used in this study (S3 Appendix).

### Meta-circles

Analysis was done to determine if non-glyphosate using areas (e.g. containing forest, water, organic farms and residential) could be differentiated from areas of higher glyphosate use, as determined by conversations with the beekeepers. Eight meta-circles were made, comprising multiple 1-Km circular zones that were grouped as having the same general land use description (Table 2, Fig 1) and situated in grouped watersheds. These meta-circles were encircled within a computer-generated circumference (mean 1,707 hectares) that fully contained 3 to 9 circular zones of the same land-use practices (ranging from 1,256 to 2,365 hectares). In total, 41 samples were included within these eight meta-circles.

### Large-scale divisions (East-West side of island, Moku)

The island of Kauai is divided by mountainous ranges and orographic rainfall in to two different biogeographical zones [16]. The drier leeward west-side of Kauaʽi comprises the Moku of Kona, Nāpali, and Mānā for approximately 73,710 hectares, 51.3% of the island’s area, while the wetter windward east-side comprises the Moku of Puna, Koʽolau, and Haleleʽa for approximately 70,049 hectares, 48.7% of the island’s area. Moku are identified by geological and biogeographic features [19] (Fig 1).

### Statistical analysis

Data was analyzed with Microsoft Excel and Access (means, medians, S.D., S.E.M, t-tests, linear and exponential line fits). Analyse-it, a plug-in for Excel, was used for correlations and AICc line fits. TIBCO Spotfire Analyst® was used to produce the Trellis plots and non-parametric Kruskal-Wallis analysis.

## Results

### Island-wide

ELISA measured glyphosate concentrations in honey taken directly from the hive ranged from < LOQ to 342 ppb (Table 3). Sixteen (27.1%) of 59 samples had glyphosate concentrations detected over the ELISA limit of quantitation (LOQ = 15 ppb).

**Table 3:** Glyphosate concentration and percent of land use (by category) within the circular zones surrounding the hives.

Google Earth Polygon Land Use Classification								[Glyphosate]
Sample #	% Urban	% Suburbs	% Open	% Ag	% Forest	% Wetland	% Water	ppb
1	71.4%	1.1%	6.6%	3.1%	0.0%	17.6%	0.2%	< LOQ
2	0.0%	30.1%	0.0%	0.0%	67.6%	2.4%	0.0%	
3	0.0%	13.5%	0.0%	70.9%	15.5%	0.0%	0.0%	
4	31.1%	0.0%	9.0%	30.0%	29.7%	0.0%	0.3%	
5	22.6%	0.0%	13.3%	21.0%	42.8%	0.0%	0.3%	< LOQ
6	19.8%	0.0%	3.3%	76.9%	0.0%	0.0%	0.0%	80
7	0.0%	10.4%	66.5%	3.2%	19.8%	0.0%	0.0%	
8	5.5%	1.8%	0.2%	90.5%	0.0%	0.0%	1.9%	61
9	0.0%	46.6%	23.1%	1.1%	29.2%	0.1%	0.0%	
10	0.0%	6.5%	87.5%	4.4%	0.0%	0.0%	1.6%	< LOQ
11	0.0%	0.0%	4.2%	69.7%	26.1%	0.0%	0.0%	
12	0.0%	0.0%	48.2%	19.8%	32.0%	0.0%	0.0%	15
13	0.0%	30.9%	26.6%	8.6%	34.0%	0.0%	0.0%	
14	5.5%	1.8%	0.2%	90.5%	0.0%	0.0%	1.9%	342
15	15.58%	13.8%	1.4%	43.6%	23.1%	0.0%	2.6%	
16	15.3%	0.0%	0.0%	53.8%	30.1%	0.0%	0.9%	
17	0.0%	4.0%	1.8%	0.0%	94.2%	0.0%	0.0%	
18	25.4%	0.0%	74.1%	0.1%	0.0%	0.0%	0.3%	25
19	52.9%	0.0%	44.6%	2.4%	0.0%	0.0%	0.0%	< LOQ
20	5.5%	1.8%	0.2%	90.5%	0.0%	0.0%	1.9%	155
21	5.5%	1.8%	0.2%	90.5%	0.0%	0.0%	1.9%	33
22	0.0%	45.8%	3.0%	33.9%	13.3%	0.0%	4.1%	
23	0.0%	50.2%	14.8%	0.4%	34.6%	0.0%	0.0%	
24	0.0%	1.5%	11.2%	64.3%	23.0%	0.0%	0.0%	
25	6.8%	10.1%	57.1%	2.3%	23.5%	0.0%	0.3%	
26	0.0%	1.0%	6.7%	68.6%	23.7%	0.0%	0.0%	
27	18.9%	0.0%	50.5%	0.0%	29.4%	0.0%	1.2%	
28	0.0%	0.0%	47.5%	11.5%	41.0%	0.0%	0.0%	< LOQ
29	0.0%	14.4%	0.5%	75.0%	10.1%	0.0%	0.0%	
30	0.0%	7.0%	30.2%	8.7%	54.1%	0.0%	0.0%	
31	0.0%	30.1%	0.0%	0.0%	67.6%	2.4%	0.0%	
32	0.0%	30.2%	8.3%	1.8%	57.7%	0.0%	2.0%	
33	22.2%	5.4%	61.4%	0.0%	7.8%	3.2%	0.0%	
34	0.0%	11.9%	1.5%	71.9%	7.4%	7.1%	0.2%	187
35	0.0%	11.9%	1.5%	71.9%	7.4%	7.1%	0.2%	178
36	0.0%	11.9%	1.5%	71.9%	7.4%	7.1%	0.2%	172
37	5.5%	1.8%	0.2%	90.5%	0.0%	0.0%	1.9%	92
38	36.2%	0.0%	2.8%	61.0%	0.0%	0.0%	0.0%	78
39	20.7%	4.2%	44.3%	3.5%	27.4%	0.0%	0.0%	
40	0.0%	49.0%	0.0%	12.9%	38.1%	0.0%	0.0%	< LOQ
41	17.1%	0.3%	0.0%	58.9%	23.8%	0.0%	0.0%	60
42	0.0%	0.0%	1.4%	67.9%	30.7%	0.0%	0.0%	
43	0.9%	1.2%	81.8%	4.7%	11.4%	0.0%	0.0%	
44	0.0%	28.2%	0.0%	25.3%	44.2%	0.0%	2.3%	< LOQ
45	0.0%	3.2%	0.0%	0.0%	95.5%	0.0%	1.3%	
46	6.8%	56.2%	0.0%	0.0%	37.0%	0.0%	0.0%	< LOQ
47	0.0%	0.0%	0.0%	19.5%	80.5%	0.0%	0.0%	
48	19.7%	0.0%	10.2%	16.3%	50.2%	0.0%	3.6%	292
49	20.0%	49.6%	0.0%	0.0%	29.0%	0.0%	1.3%	
50	0.0%	1.5%	0.0%	50.4%	48.1%	0.0%	0.0%	
51	0.0%	16.4%	0.0%	0.0%	83.6%	0.0%	0.0%	
52	19.0%	4.2%	2.2%	51.6%	21.4%	0.0%	1.6%	
53	19.0%	4.2%	2.2%	51.6%	21.4%	0.0%	1.6%	
54	19.0%	4.0%	2.2%	47.3%	24.1%	0.0%	3.4%	
55	18.0%	4.2%	2.2%	42.6%	31.2%	0.0%	1.8%	
56	19.0%	4.0%	2.2%	47.3%	24.1%	0.0%	3.4%	
57	15.6%	13.8%	1.4%	43.6%	23.1%	0.0%	2.6%	27
58	19.0%	4.0%	2.2%	47.3%	24.1%	0.0%	3.4%	
59	25.4%	0.0%	74.1%	0.2%	0.0%	0.0%	0.3%	95

Calculations of mean concentrations were done in two manners: using all sample ELISA data (N = 59, mean = 33.5 ppb, standard error of the mean, S.E.M. = 9.3) or for only those samples with ELISA values greater than the LOQ (N = 16, mean = 118.3, S.E.M. = 24.0).

### Spatial and temporal variations at hive sites

Six separate sites had samples taken from multiple unique hives on those sites. At two of these six sites (Samples # 52, 53; 54, 56, 58), all hives had no glyphosate detected. At three of these six sites (Samples # 18, 59; 8, 14, 20, 21; 34, 35, 36), all hives had glyphosate > LOQ. At one site (Samples # 55, 57), only one hive had detectable glyphosate (Sample # 57) (27 ppb), while the other hive had none detected.

An extremely large feral beehive sampled in 2013 had 92 ppb glyphosate (Table 3, Sample # 37). In 2015, this site had four samples taken from widely spaced parts of the hive (Samples 8, 14, 20 & 21). Analysis yielded values ranging from 33 ppb to 342 ppb (mean = 147.7 ppb, S.E.M. = 69.7 ppb).

Two different sites were sampled in 2015 and again in 2016. Each of these two sites had multiple hives. Both sites showed an increase in concentration levels over time (0 ppb to 27 ppb for samples 55 & 57; 25 ppb to 95 ppb for Samples 18 & 59).

Of the store-bought samples (Table 4 and Table A in S4 Appendix), 33.3% of those from Kauaʽi had glyphosate residue > LOQ (mean = 41 ppb, S.E.M. = 14.2).

**Table 4:** Concentration and percentage of glyphosate detected in store-bought honey. Samples originated from three Hawaiian islands and international blends. Samples categorized as Organic or Non-Organic.

Category			Samples N	> LOQ %	> LOQ Mean ppb
Location	** **	** **	** **	** **	** **
	Hawaii	Island:			
		Kauaʽi	15	33.3	41.0
		Hawai'i	4	25.0	16.4
		Molokai	2	0	NA
	International		5	40.0	51.5
Type					
	Organic		5	20.0	30.6
	Non-Organic		21	33.3	42.0

### East-West side of island

Presence and concentration of glyphosate residues were mapped with respect to ancient Hawaiian biogeographical and management land divisions (Moku) [19]. When all 59 samples were analyzed, there was a higher glyphosate concentration (mean = 61.6 ppb, N = 31, S.E.M. = 16.2) (Table 5 and Tables B and C in S4 Appendix) in honey from the leeward western half of Kauaʽi versus the windward eastern half (mean = 2.4 ppb, N = 28, S.E.M. = 0.9). Mean values between the western and eastern sides are different (t-test, p = 0.001, df = 57) (Table D in S4 Appendix).

**Table 5:** Glyphosate concentration by side of island and the six Moku. All 59 sample values used. Napali Moku had no samples (“ns”).

Moku	Glyphosate Mean ppb	Median	S.D.	S.E.M.	Count
Windward:					
Koolau	0	0	0	0	10
Puna	2.5	0	5.1	1.7	9
Halelea	4.1	0	6.3	2.1	9
Totals	2.41	0	4.9	0.92	28
Leeward:					
Kona	53.9	11.7	80.9	14.8	30
Mana	292.2	292.2	na	na	1
Napali	ns	na	na	na	ns
Totals	61.61	13	90.3	16.2	31

If only glyphosate values > LOQ are used (N = 16), the western Moku had 15 samples, 48.4% of which had glyphosate > LOQ (mean = 125.1 ppb). The eastern Moku had only 1 sample over the LOQ (3.6%). This sample value (15.2 ppb) is just greater than the LOQ.

**Fig 3:**
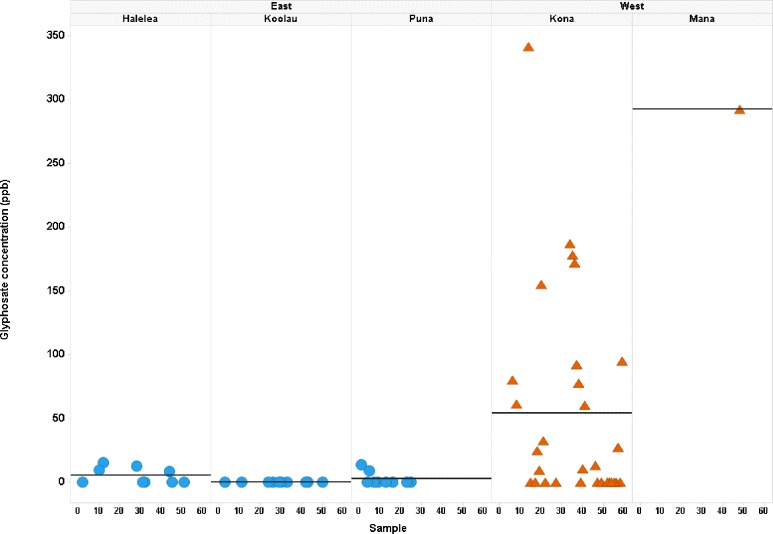
Glyphosate concentrations across samples by side of island and within each Moku. Mean glyphosate (ppb) is shown by the horizontal line for each Moku. Side of the island and Moku names are listed at the top of the plot. Samples from the western Moku are shown as orange triangles and eastern Moku as blue circles.

A Trellis plot was made showing the glyphosate concentration across samples, grouped by side of island and by Moku. When all 59 samples are plotted, there is a clear pattern of the higher glyphosate concentrations in the western Moku vs the eastern Moku (Fig 3). No samples were collected from the remote western Moku of Napali.

### Moku

Moku differed greatly in the and mean concentration of glyphosate in honey (Table B in S4 Appendix). Puna and Ko’olau Moku had no samples >LOQ and Halele’a had only one >LOQ. No samples were collected from remote Napali and only one sample from Mana. Concentrations from the west side Kona Moku were different from the three east-side Moku (p < 0.003) (Table E in S4 Appendix).

Since it is not known if these samples are from a normally distributed population, a non-parametric Kruskal-Wallis test was performed. This test confirmed the above parametric tests that glyphosate distributions were different depending upon the side of the island and the Moku (p = 0.0008 and 0.004, respectively) (Table F in S4 Appendix).

Source location of honey purchased from merchants on Kauaʽi was obtained from the label and discussions with vendors. Percentage of samples with glyphosate residue > LOQ and mean concentrations of glyphosate differed among Moku sampled (Table 6 and Table A in S4 Appendix). Area with the greatest percentage of samples with glyphosate was in the agricultural district of Kona on the west side of the island. This is the same trend seen as with the hive samples (Fig 3).

**Table 6:** Prevalence and concentration of glyphosate in Kauai honey from store-bought samples.

Moku	All samples N	> LOQ N	> LOQ % total	> LOQ mean	> LOQ SEM
Puna	6	1	16.7	15.0	na
Koolau	5	1	20.0	61.8	na
Kona	4	3	75.0	43.1	22.2

### Circular zones and land use polygons

Land use within an area of 1 Km radius around each of the hives was determined using Google Earth Pro™ (GEP) (N = 59 hives from Kauaʽi). These circular zones were divided into single land use polygons and the total meter^2^ coverage for each of the seven land types was calculated. The percent of the total allocated to each of the seven land use types of each site was summarized with the glyphosate concentration found in the samples from that site (Table 3).

AICc analysis was performed to determine correlations between presence of glyphosate in honey and various land uses. Non-zero glyphosate data (N = 23) was used for these analyses. The exponential model for land use and glyphosate was chosen, as it has the highest correlation and strongest AICc values, compared with other line fits (Table G in S4 Appendix). Agriculture land use in the immediate 1 Km radius vicinity of the hive showed the highest positive correlation with glyphosate concentration (Table 7, R^2^ = 0.594) and the strongest AICc compared with the other land use categories. Open, Suburbs, Urban, and Forest land use all showed weak negative correlations (negative Parameter Estimates) between land use and glyphosate concentration. Wetland and Water land use showed very weak positive correlations. The negative correlations (e.g. Forest) is due to these land use types not being independent variables; rather, they are multicollinear (Figure A in S4 Appendix).

**Table 7:** Correlation of glyphosate concentration (ppb) in honey samples and the percent land use.

Land Use	R^2^	AICc	SE of fit (RMSE)	Parameter estimate	95% CI	95% Cl	SE	p-value	Exponential Equation
Agriculture	0.594	-8.664	0.784	2.552	1.594	3.511	0.461	0.000	Y = 12.58 * 12.84 ^x^
Forest	0.326	2.967	1.010	-3.977	-6.572	-1.383	1.247	0.004	Y = 65.24 * 0.01874 ^x^
Open	0.123	9.030	1.152	-1.465	-3.242	0.311	0.854	0.101	Y = 50.03 * 0.231 ^X^
Suburbs	0.086	9.973	1.176	-2.276	-5.638	1.087	1.617	0.174	Y = 46.98 * 0.1027 ^X^
Urban	0.049	10.897	1.200	-1.422	-4.274	1.430	1.371	0.311	Y = 47.01 * 0.2412 ^X^
Wetlands	0.017	11.660	1.220	3.659	-9.110	16.427	6.140	0.558	Y = 36.23 * 38.8 ^X^
Water	0.011	11.796	1.224	13.180	-44.017	70.377	27.504	0.637	Y = 34.83 * 5.296e+05 ^X^

**Fig 4:**
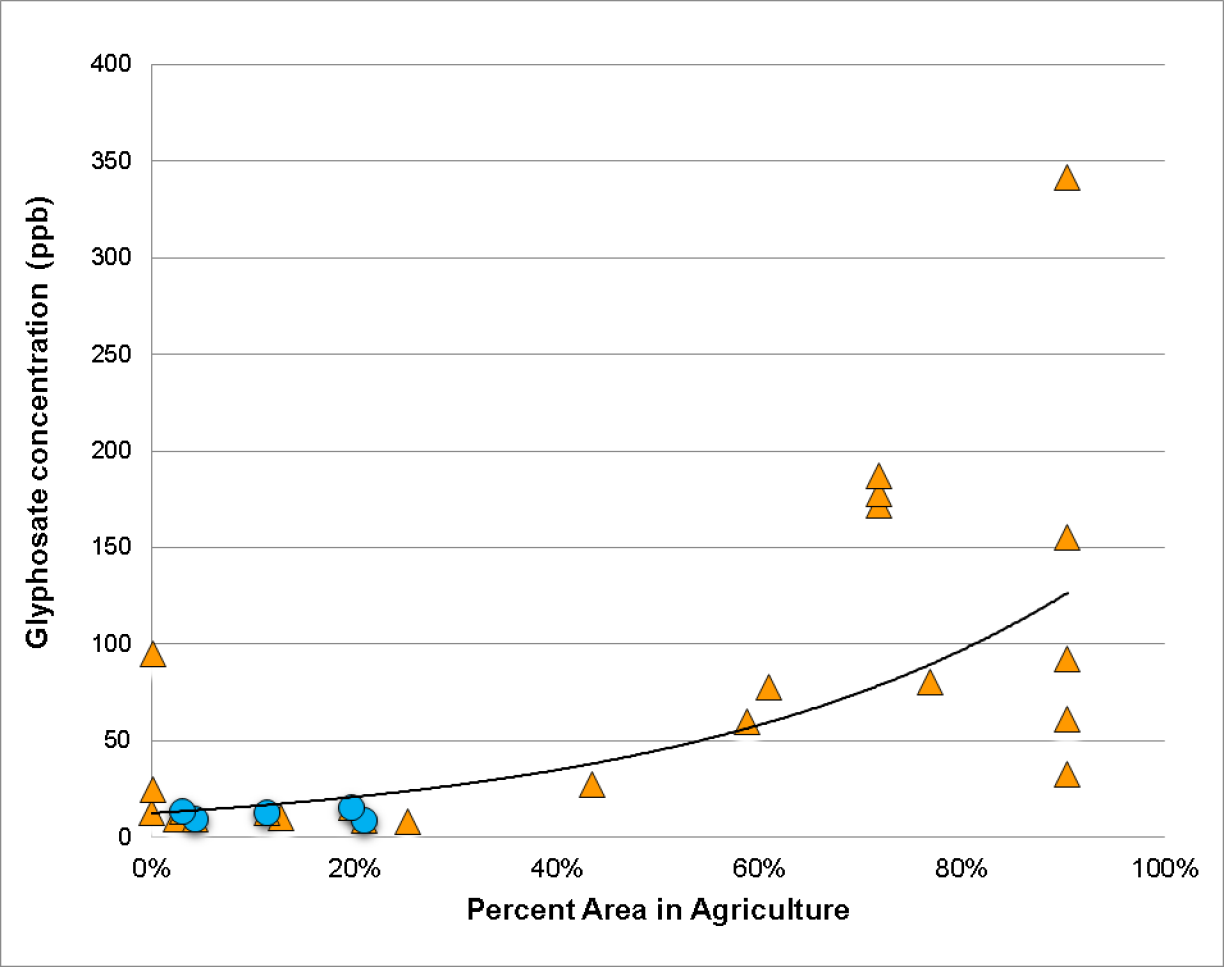
Glyphosate concentration versus the percent land use in agriculture (N = 23). Samples from the western Moku are shown as orange triangles and eastern Moku as blue circles. Exponential fit is Y = 12.6 e^12.8X^, R^2^ = 0.594.

Concentration of glyphosate in honey was plotted versus the percent land use in agriculture. Samples with non-zero glyphosate were used (N = 23). Fig 4 shows that the higher glyphosate concentrations are correlated with sites that have high percent agriculture land use (> 60% agriculture).

The hives in the western Moku (orange triangles) have a strong correlation with higher glyphosate when there is higher percent land use as agriculture. Hives in the eastern Moku (blue circles) had very low glyphosate, even with 60% to 80% of the area in agriculture (Table 3).

### Meta-circles

In order to expand land use to watersheds or larger areas, meta-circle analysis was done on eight clusters of circular zones situated all around the island (Fig 1). They comprise similar environments. Discussions with beekeepers were used to develop a general description of each meta-circle (Table 8) as to predominant land use and glyphosate use.

**Table 8:** Meta-circle composition, mean glyphosate concentration, and percent prevalence. Meta-circle # corresponds to Fig 1.

Meta-circle #	Meta-circle	Number of Circular Zones	General Description	Composite % land use	Composite land use type	Mean ppb	% > LOQ
1	Kīlauea	5	Rural, Suburbs	72.0%	Open, Forest	< LOQ	0%
2	Moloaʽa	6	Organic farming	89.2%	Agriculture, Forest	< LOQ	0%
3	Kapaʽa	4	Suburbs	82.0%	Open, Forest, Suburbs	< LOQ	0%
4	Līhuʽe	4	Urban, open, agriculture	87.7%	Urban, Agriculture, Forest	< LOQ	0%
5	Kōloa	9	Suburbs, golf, resort	74.7%	Agriculture, urban, Forest	16.3	33%
6	Lāwaʽi	5	Suburbs	82.9%	Forest, Suburbs, Open	< LOQ	0%
7	Ag. 1	3	Large scale agriculture	71.9%	Agriculture	179.0	100%
8	Ag. 2	5	Large scale agriculture	90.5%	Agriculture	136.6	100%

The percent of each of the seven types of land use was calculated for each circular zone in each meta-circle (Table 3 and Table H in S4 Appendix). Then the mean percent of each type of land use was calculated for each meta-circle. The highest percent land use was used to describe the meta-circle, if that land use type was at least 70%. If it was less than 70%, then a composite was used; the second highest type of land use was added to the highest land use type. This process was repeated until the composite land use designation comprised at least 70% of the meta-circle. This composite description is shown in Table 8, in the column “Composite land use type”.

The mean concentration of glyphosate in honey was calculated using all samples within each meta-circle (N = 48 samples total). The percentage of samples which had glyphosate > LOQ was also calculated (N = 16 total). Only three meta-circles had significant glyphosate residues and all were in areas on the western side of Kauaʽi. The two meta-circles with the most glyphosate, Ag. 1 and Ag. 2, were in areas of large scale agriculture use. The Koloa meta-circle had some agricultural use and contained the circular zones with large amounts of golf courses and or highway present, as discussed below.

**Fig 5:**
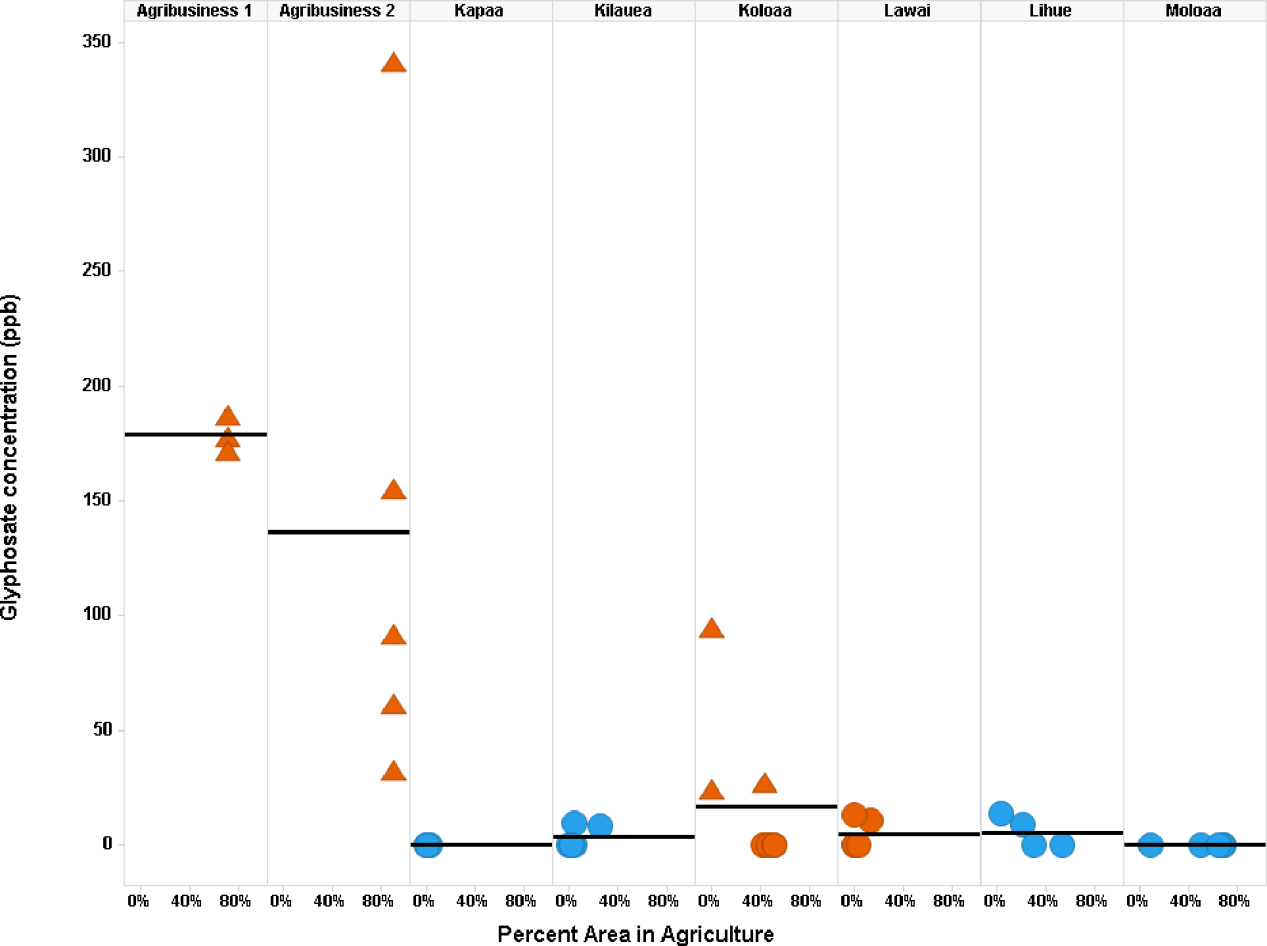
Glyphosate concentrations across samples within each meta-circle. Mean glyphosate (ppb) is shown by the horizontal line for each meta-circle. Meta-circle names are listed at the top of the plot. Samples from the western Moku are shown as orange and eastern Moku as blue. Samples with glyphosate > LOQ are shown as triangles, while those < LOQ are as circles.

A Trellis plot was made to show glyphosate concentration across samples, grouped by meta-circle (Fig 5). Within each meta-circle, samples are plotted versus the percentage of agriculture for that sample. There is a clear pattern of the higher glyphosate concentrations for samples in the western meta-circles (orange) vs samples in the eastern meta-circles (blue). The samples with glyphosate > LOQ (triangles) are also all in the western meta-circles, while the eastern meta-circles all have glyphosate <LOQ (circles) (Fig 5).

### Golf courses and highways

A smaller specific land use, golf course, was identified from GEP images, but was subsumed in the “Developed Open” C-CAP category (Table 2). There were only eight circular zones which encompassed golf course(s) and all had glyphosate residues in honey (Table 9A). Percent area in golf course varied from 1.2% to 16.2%. Three of those samples (samples #34, 35, 36) were from different hives on the same farm and were also associated with high percent (> 70%) agricultural land use. Two hives with the highest percent land use as golf course (samples # 18 and #59) were from the same residence with very low agricultural land use.

**Table 9:** (A) 8 samples with highest % area Golf; (B) 6 samples with highest Km highway present.

A				
Sample #	Glyphosate ppb	% Ag	% Golf	Km Highway
34	187	71.9%	1.2%	1.1
35	178	71.9%	1.2%	1.1
36	172	71.9%	1.2%	1.1
19	10	2.4%	1.6%	3.4
1	14	3.1%	4.8%	2.0
28	13	11.5%	13.7%	2.0
18	25	0.1%	16.2%	4.7
59	95	0.2%	16.2%	4.7
**B**				
55	0	42.6%	0.0%	4.6
57	27	43.6%	0.0%	4.6
59	95	0.2%	16.2%	4.7
18	25	0.1%	16.2%	4.7
52	0	51.6%	0.0%	4.7
53	0	51.6%	0.0%	4.7

Major highways were identified as another small specific land use. These were subsumed under the Urban and Suburban/Rural categories (Table 2). Portions of highways were contained within 76% of the circular zones (Table I in S4 Appendix). Those in the top 10% of cumulative length of highway (> 4.6 Km) had three samples with glyphosate > LOQ (25 to 95 ppb) (Table 9B). Frequent spraying of golf courses and highways may explain the presence of glyphosate (> LOQ) in samples # 18, 57, and 59.

## Discussion

The presence of glyphosate residue in honey samples taken directly from the hive has been shown to correlate with areas that geospatial analysis has identified as comprised mainly of large-scale mono-crop agriculture. This suggests both a source and a pathway whereby pesticides migrate from site of use into other areas. Glyphosate residue >LOQ was found in 27.1% of the hives and 33.3% of store bought honey from Kauai, lower than the 59% in store bought honey from around the world [1]. With hive-collected honey, geospatial analysis was able to further identify: which side of the island (west), which Moku (Kona and Mana), which areas (agriculture meta-circles), and most specifically which land use (agriculture) had the greatest prevalence and greatest concentration of glyphosate in honey.

Purchased samples from the other Hawaiian islands had lower mean concentrations and a smaller percentage contaminated than those from Kauai. The mean concentration of glyphosate from international samples purchased on Kauai was 51.5 ppb, similar to the 64 ppb in Rubio [1]. Samples from Brazil and a sample from a blend of USA and Argentina approximated values reported earlier, while the blend from Brazil, Mexico and Uruguay did not [1].

One of five Kauai purchased samples (20%) labeled organic had glyphosate residues > LOQ (mean 30.6 ppb) compared to 45% (mean 50 ppb) reported elsewhere [1]. This supports supposition of some migration of pesticides from areas of application to organic farms. The twenty-one Kauai samples not labeled as organic had both a higher occurrence (33.3%) and higher mean concentration (42.0 ppb) of glyphosate than the organic labeled samples, suggesting application of glyphosate near the hives. Honey from traditional agriculture sites around the world had 62% with glyphosate >LOQ and mean 66 ppb [1], expressing widespread use of glyphosate in agriculture.

The actual process of how Kauai bees obtained, carried and processed glyphosate is not known and was not addressed in this study, but is discussed elsewhere [13,14]. As honey was obtained directly from the hive using clean vials, this eliminated the possibility of contamination occurring during processing. Each sample was unique to a single hive, not blended from various sites. A survey of beekeepers confirmed that their hives did not get sprayed with glyphosate. Uptake could have occurred if the bees themselves got sprayed while foraging, if flowers frequented by the bees contained glyphosate from either direct spraying or aerial drift, or if water that the bees drank on plants or on the ground was contaminated in some way. In all cases, contamination could have occurred at a distance from the hive. Geospatial analysis allowed the determination that within a 1 Km radius of the hive, glyphosate contamination was most closely associated with large scale agriculture. The proximity of golf courses and highways were also associated, but to a lesser degree. General land use changes and landscape composition may have indirect detrimental effect on bee fitness, although the association between pesticide and landscape composition was not investigated].

The presence of both restricted use pesticides and glyphosate in bee pollen and honey, even at very low levels, identifies an important pathway whereby pesticides migrate from site of application to the hive and into the human food supply [12–14, 26]. Geospatial analysis can help honey producers estimate spatial pesticide exposure risks inherent in intensive agriculture. When bees are used for commercial large-scale crop pollination, hive placement can be optimized so that the bee colonies are not seriously impacted by pesticides that the bees must endure while foraging [26–27]. Linking spatial and temporal dynamics of flowering crops, agri-environmental schemes, and pesticide applications would lead to better understanding of environmental risk assessment, management of pollination services, and protecting biodiversity [26–28].

## Supporting information

S1 Appendix Abraxis technical bulletin. (DOCX)

S2 Appendix ELISA verification with mass spectrometry. (DOCX)

S3 Appendix Geospatial analytical method comparison. (DOCX)

S4 Appendix Glyphosate data from Kauai hives and store-bought honey. (DOCX)
