# Supplementary material for: Glyphosate residue concentrations in honey attributed through geospatial analysis to proximity of large-scale agriculture and transfer off-site by bees
Source: PLoS One. 2018 Jul 11;13(7):e0198876. doi: 10.1371/journal.pone.0198876 (PMC6040695; doi:10.1371/journal.pone.0198876)
Supplement: S1 Appendix — (DOCX) [file pone.0198876.s001.docx]

**Supplemental Information 1. Appendix.** Abraxis Technical Bulletin

**Glyphosate in Honey and Corn Syrup**

**Sample Preparation**

1. **Intended Use**

For the detection of Glyphosate in honey and corn syrup.

1. **Sensitivity**

0.015 ppm in matrix

# Materials and Reagents Required

Analytical balance

Microcentrifuge tubes

4 mL glass vials with Teflon-lined caps

Disposable pipettes

Micropipettes with disposable plastic tips

Vortex mixer

Microcentrifuge

Timer

Plate shaker or Micro-well plate holder with insert retainer for vortex mixer

1 N Hydrochloric Acid (HCl)

Glyphosate sample diluent

Abraxis Glyphosate Plate ELISA Kit

# Notes and Precautions

This procedure is intended for use with honey and corn syrup (light and dark). Other matrices should be thoroughly validated before use with this procedure.

Hydrochloric Acid must be handled with care. Wear appropriate protective clothing (gloves, glasses, etc.). Avoid contact with skin and mucous membranes. If contact occurs, wash with copious amounts of water and seek appropriate medical attention.

Due to the viscous nature of the prepared samples, the microtiter plate should be placed on a plate shaker or vortex mixer fitted with a micro-well plate holder adapter for the incubations with the antibody and conjugate solutions. This will allow for the appropriate mixing of all reagents in the microtiter wells.

# Sample Preparation Procedure

5.1 Weigh 0.5 g of sample into an appropriately labeled microcentrifuge tube.

5.2 Add 0.5 mL of 1 N HCl. Vortex for 2 minutes.

5.3 Add 3.96 mL of Glyphosate Diluent to a clean, appropriately labeled 4 mL glass vial. Add 40 µL of the acid-treated sample (from step 5.2) to the Glyphosate Diluent in the vial (1:100 sample dilution). Vortex. This will then be analyzed as sample, see *Derivatization of Standards, Control, and Samples* in the Reagent Preparation section of the Glyphosate Plate ELISA Kit user’s guide.

# Evaluation of Results

The Glyphosate concentration in the samples is determined by multiplying the ELISA results by a factor of 200.

Samples showing a concentration lower than standard 1 (0.075 ppb) should be reported as containing < 15 ppb of Glyphosate. Samples showing a higher concentration than standard 5 (4.0 ppb) can be reported as containing > 800 ppb of Glyphosate or diluted further and re-analyzed to obtain an accurate quantitative result.

# Performance Data *Recovery*

Honey samples were spiked with various amounts of Glyphosate, prepared as described above, and then derivatized and assayed using the Glyphosate Plate Assay. Average recovery was 113%.

Corn syrup samples (light and dark) were spiked with various amounts of Glyphosate, prepared as described above, and then derivatized and assayed using the Glyphosate Plate Assay. Average recovery was 104%.

# Assistance

For ordering or technical assistance contact:

Abraxis LLC

54 Steamwhistle Drive

Warminster, PA 18974

Tel.: (215) 357-3911

Fax: (215) 357-5232

Email: info@abraxiskits.com

Web: www.abraxiskits.com

041714
