# Supplementary material for: Glyphosate residue concentrations in honey attributed through geospatial analysis to proximity of large-scale agriculture and transfer off-site by bees
Source: PLoS One. 2018 Jul 11;13(7):e0198876. doi: 10.1371/journal.pone.0198876 (PMC6040695; doi:10.1371/journal.pone.0198876)
Supplement: S2 Appendix — (DOCX) [file pone.0198876.s002.docx]

**Supplemental Information 2. Appendix.** ELISA verification with mass spectrometry.

To verify ELISA techniques for measuring glyphosate in a honey matrix (LOQ of 15 ng/g, 15 ppb) honey remaining in 14 vials from the Batch 2 samples analysed with ELISA were sent to Quality Services International GmbH (QSI), (Bremen, Germany) for analysis of herbicide residue by gas chromatography/mass spectrometry (GC/MS/MS) and/or liquid chromatography mass spectrometry (LC/MS/MS) methods (QSI method # 88505) with a LOQ of 0.01 mg/kg (10 ppb) (Table A). All concentrations derived from ELISA were used in analysis, however QSI did not report readings for levels <10 ppb, so a value of zero was assigned for data analysis.

**Table A. Glyphosate concentrations in honey matrix using either ELISA techniques or LC/GC/MS/MS techniques.** Bold face numbers exceeded both techniques’ LOQ and were plotted separately.

|  | Glyphosate ppb | Glyphosate ppb |
| --- | --- | --- |
| Sample # | ELISA | QSI |
| 1 | 13.6 | 17 |
| 5 | 8.8 | 10 |
| 6 | **80.2** | **63** |
| 7 | 0 | 0 |
| 10 | 9.2 | 0 |
| 12 | 15.2 | 0 |
| 14 | **341.6** | **330** |
| 16 | 0 | 0 |
| 18 | **24.6** | **21** |
| 19 | 9.6 | 12 |
| 25 | 0 | 0 |
| 27 | 0 | 0 |
| 28 | 12.6 | 0 |
| 35 | **178.0** | **190** |
| LOQ= | 15 | 10 |

Results for all 14 samples analysed by both methods correlated well (Fig A). Standard error of y for each x-value is 8.6 ppb. Only 4 samples had both the ELISA and LC/GC/MS/MS values over their respective LOQ, but the correlation coefficient remained high (Fig A).

**Fig A. Correlation of glyphosate concentration in honey split-samples using ELISA and LC/GC/MS/MS techniques.** Linear fits: Y = 0.99 x – 3.1, R^2^ = 0.993 (N = 14; black and blue circles); Y = 0.99 x + 6.1, R^2^ = 0.994 (N = 4; blue circles).

Although sample size was small, a correlation coefficient R^2^ = 0.99 supports the ELISA tests for accuracy, in addition to the use of blank and standards within each test run [1]. Comparison of ELISA techniques for monitoring glyphosate with chromatography-mass spectrometry have consistently found high correlations between the techniques in tests of various matrices, e.g. water [2, 3], animal urine and animal tissues [4]. The use of Abraxis methods of ELISA determination of glyphosate in honey is well substantiated.

References

1. Rubio F, Guo E, Kamp L. Survey of Glyphosate Residue in Honey, Corn, and Soy Products. J Environ Anal Toxicol. 2014; 5:249. doi: 10.4172/2161-0525.1000249

2. Byer JD, Struger J, Klawunn P, Todd A, Sverko E. ELISA testing for Low cost monitoring of glyphosate in surface waters using the ELISA method: an evaluation. [Environ Sci Technol. 2008; 42(16): 6052-6057](http://dx.doi.org/10.1021/es8005207)

3. Josep S, Kantiani, Llorca M, Rubio F, Ginebreda A, Fraile J, Garrido T, Farré M. Determination of glyphosate in groundwater samples using an ultrasensitive immunoassay and confirmation by on-line solid-phase extraction followed by liquid chromatography coupled to tandem mass spectrometry. Anal Bioanal Chem DOI 10.1007/s00216-011-5541-y

4. Krüger M, Schledorn P, Schrödl W, Hoppe HW, Lutz W, et al. 2014; Detection of Glyphosate Residues in Animals and Humans. J Environ Anal Toxicol 4:210. doi: 10.4172/2161-0525.1000210

5. Krüger M, Schrödl W, Neuhaus J, Shehata AA. 2013; Field Investigations of Glyphosate in Urine of Danish Dairy Cows. J Environ Anal Toxicol 3:186. doi: 10.4172/2161-0525.1000186
