# Supplementary material for: Glyphosate residue concentrations in honey attributed through geospatial analysis to proximity of large-scale agriculture and transfer off-site by bees
Source: PLoS One. 2018 Jul 11;13(7):e0198876. doi: 10.1371/journal.pone.0198876 (PMC6040695; doi:10.1371/journal.pone.0198876)
Supplement: S3 Appendix — (DOCX) [file pone.0198876.s003.docx]

**Supplemental Information 3. Appendix***.* Development, application and comparison of two means for quantifying current land use practices within 1 Km radius of a bee hive.

**Geospatial analysis was performed in ArcGIS 10.5 on two separate Habitat datasets**:

1. Coastal Change Analysis Program (C-CAP) High Resolution Land Cover (1-4 meter resolution). Derived from high resolution imagery and analyzed according to the Coastal Change Analysis Program (C-CAP) protocol to determine land cover.^[[1]](#endnote-1)^
2. Vector polygons digitized in Google Earth Pro™ (GEP) using Digital Globe™ (DG) images (2013-2014) of 30-50 cm resolution as a base layer.

**Coastal Change Analysis Program (C-CAP)**

C-CAP analysis was conducted in January of 2016. Data downloaded was produced at a 1-4 meter resolution and utilized 35 full or partial WorldView2 multispectral scenes and the 2005 high-resolution Kauai C-CAP data set. The imagery and base classification were included in a multi-step semi-automated change detection process to extract land cover features in the 2010 imagery.^[[2]](#endnote-2)^ Habitat within this dataset is classified into one of 21 different habitat classifications using a 2.5 meter cell size.

In order to extract out raster cells within the 2 kilometer boundary (1 Km radius) per hive site, the data set was masked using a vector dataset. This dataset was created by plotting each of the 38 hive sites in ArcGIS using their UTM location. Locations were converted into a point shapefile and then buffered by 1 km to create the 2 kilometer boundary polygon. Individual polygons were dissolved into one record to create the Mask to extract out pixels of the CCAP raster. Masking a raster using a vector is similar to the “Clip” geoprocessing routine done between two vector datasets. A vector representing an outline of the island was used to further mask the raster, removing pixels that were beyond the coastline, seemingly representing ocean (Fig A, B).

In order to quantify the percentage of habitat within each hive site boundary area (buffer a.k.a. circular zone), the raster pixels were converted into a polygon feature class (vector) for vector geoprocessing. This polygon conversion resulted in 26,176 records/polygons, representing 26,176 cells within the original Raster dataset residing in the hive site boundary area. The “Intersect” geoprocessing tool was used next to assign to each record the corresponding hive site number it fell within. Habitat codes were reclassified, reducing the number of habitats considered by the analysis to seven land use categories. These were used in identifying the candidate habitats bees are believed to be foraging. Using the “Dissolve” geoprocessing tool, the habitat polygons were dissolved by Hive Site and reclassified Habitat Code, and the results stored in a geodatabase so that the area for each habitat could be reported using the Shape Area field. Totals for the amount of habitat polygon cells residing within each hive site boundary were then summed and the percentage for each habitat within the boundary calculated*.*

**Fig A ArcGIS 10.5 geoprocessing tools: Clip, Dissolve and Intersect.**


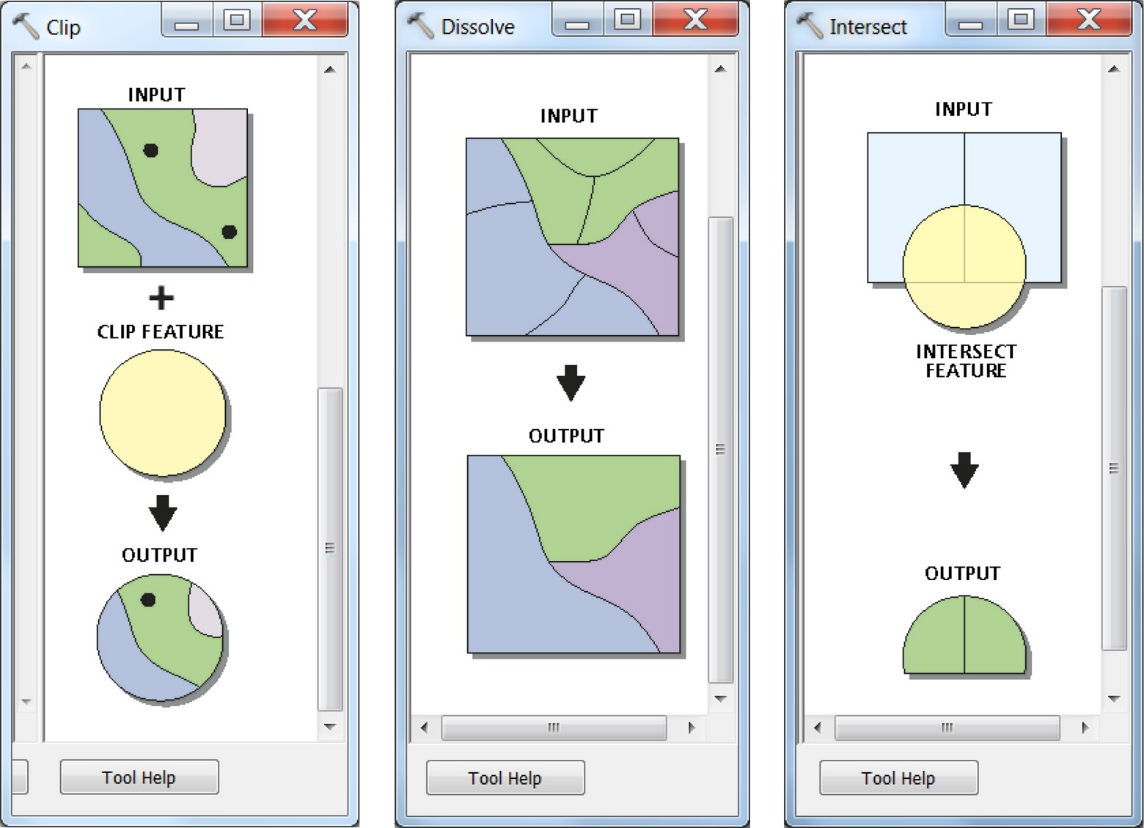


*
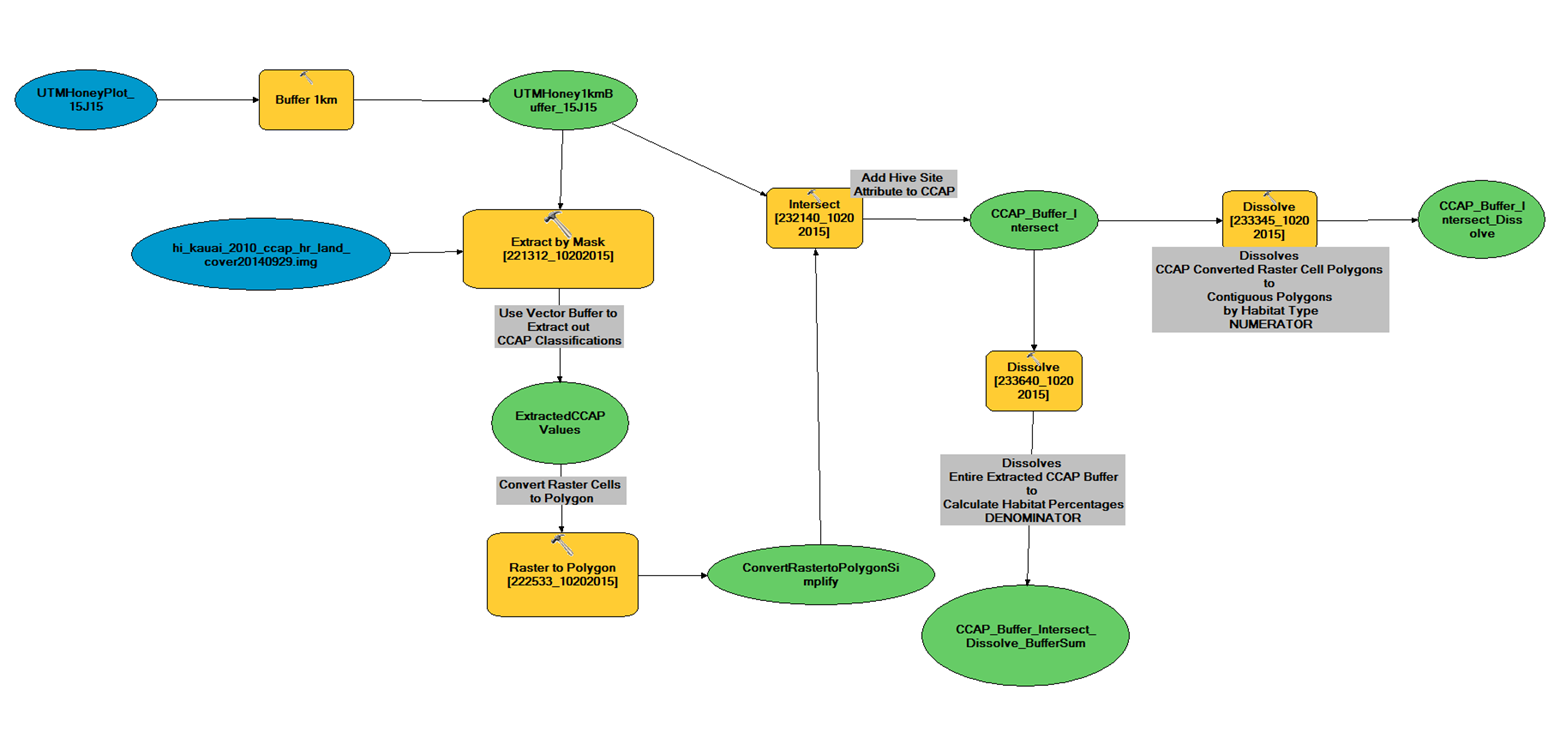
*

**Fig B. Schematic of geoprocessing tools used to improve calculations of polygon areas for the C-CAP dataset.**

**Google Earth Pro™ (GEP)** Digitizing in a Geographic Information System is the process of converting geographic data from a hard-copy or scanned image into a vector dataset by tracing features; features are captured in coordinates and stored as either a point, line or polygon vector dataset.^[[3]](#endnote-3)^ For this analysis, “heads up digitizing” in GEP was used to create discrete habitat polygons based on the reclassified habitats in the C-CAP analysis. Polygons created in GEP were stored as a KML/KMZ file, imported into ArcGIS 10.5 and converted into a feature class residing in a geodatabase so that areas of each habitat polygon could be calculated in square meters.

Upon importing the polygons from Google Earth, numerous topological errors were discovered in the polygons themselves, the most pervasive being knots, loops and slivers. These occur when “…the digitizer has an unsteady hand and moves the cursor or puck in such a way that the line being digitized ends up with extra vertices and/or nodes”.^[[4]](#endnote-4)^ Knots and loops result when a line forming a boundary of a polygon folds back on itself, creating small polygon like geometry known as “weird polygons”. ^[[5]](#endnote-5)^

Polygon features are **enclosed areas** created from a series of vertices that are connected with a continuous line traveling in one direction whereby the starting and ending point are coincident (Fig C).^[[6]](#endnote-6)^ Because the depiction of the polygon begins with a start point and travels in one direction, the resulting geometry of the polygon means the GIS can interpret what area is ‘right’ as opposed to ‘left’ of the boundary, as well as what area is enclosed by the boundary of the entire polygon; when a knot or loop occurs, the topology of the polygon actually becomes confounded due to the extra node between them. As a result, right and left sides of the boundary violates the topological relationship of the polygon itself, preventing performance of common geoprocessing tasks (clip, intersect and dissolve).

*
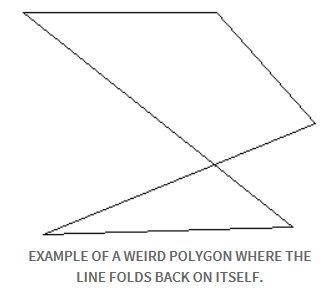
*

Fig C from <https://www.gislounge.com/digitizing-errors-in-gis/>

Another confounding topological error involves slivers. “Slivers are gaps in a digitized polygon layer where the adjoining polygons have gaps between them or where the two adjacent polygons overlap in error”.^[[7]](#endnote-7)^ This can inadvertently lead to areas among the polygons to have conflicting attributes as to what habitat the slivers represent (Fig D).

*
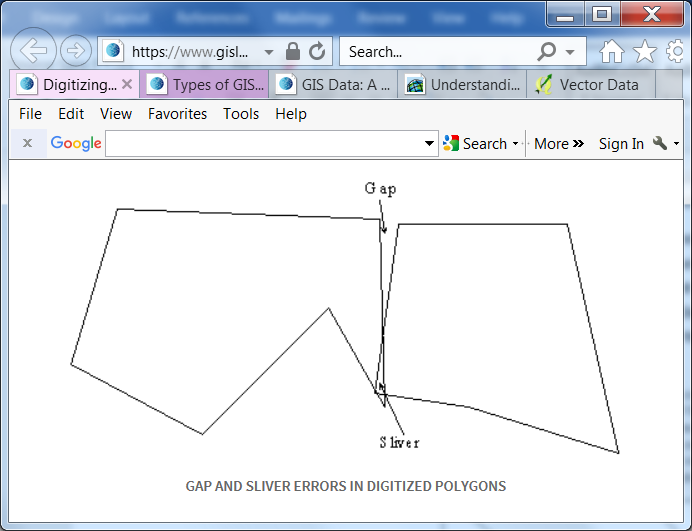
*

Fig D from <https://www.gislounge.com/digitizing-errors-in-gis/>

Manual digitizing habitat polygons is time consuming and tedious. For this analysis, and to reduce anticipated issues related to slivers, it was decided early on in the digitizing process that the largest habitat within a circular zone could be left un-transcribed and the void filled utilizing geoprocessing tools in ArcGIS. Unanticipated topological inconsistencies related to knots and loops however prevented these geoprocessing tools to be run and thus required that topology of all individual polygons to be inspected and corrected.

“Topology in GIS is generally defined as the spatial relationships between adjacent or neighboring features”^[[8]](#endnote-8)^ Planar topology requires that [intersection](https://en.mimi.hu/gis/intersection.html)s for lines and [polygon](https://en.mimi.hu/gis/polygon.html)s in a [digital data](https://en.mimi.hu/gis/digital_data.html) [layer](https://en.mimi.hu/gis/layer.html) is enforced and that no two lines or polygons cross.^[[9]](#endnote-9)^ This process involves removing twisted or self-intersecting polygons (i.e. knots and loops) so as to ensure that the “inside” of the polygon is on the correct side of the boundary.^[[10]](#endnote-10)^ It also includes removing overlaps (i.e. slivers) found by intersecting each polygon with all other polygons.^[[11]](#endnote-11)^

Tools from ET Geowizards 11.3 were used to correct planar topology, rigorously testing and correcting for topological correctness and verifying the spatial relationships between neighboring polygons. Eight circular zone sites were chosen to validate the hand-drawn polygon designations and to determine if the process would improve calculations of polygon areas. Overestimation of the initial polygons varied by only 2.5% (n=51, t-test no significant difference in paired data p=.875)

Once the topology of the GEP dataset was reconciled, the “Intersect” geoprocessing tool was used to fill voids and assign a habitat code. The dataset was then “clipped” using an “island” polygon to remove those portions of the circular zone that extended past the coastline. Since there were multiple polygons representing a given habitat within a circular zone, the “dissolve” tool was used to consolidate records so that percent habitat calculations could be completed for each circular zone.

Total area of each habitat type for each 1 km hive site circular zone was summed and the percentage calculated (Table 2 in text). Each circular zone comprised approximately 314.16 hectares, unless ocean surface area was removed. A total of 18,872 hectares of land area was classified for the vector polygon dataset. Visual ground truthing was performed to ensure images in the GEP imagery matched images on the ground.

**Comparing results between the C-CAP and GEP Datasets**

C-CAP high-resolution land cover for 2010, produced at 2.4 m resolution, was applied to the 38 sites from the 2013 and 2015 sampling and compared to the same data grouped and processed using GEP polygons. For Agriculture and Urban land-cover categories, the two methods produced similar mean values, were not significantly different (t-test), and were well correlated (TableA). For Forest, Open and Water land-cover, the mean values were significantly different.

**Table A. Comparison of means and correlations between C-CAP and GEP methods for different land use.**

|  | *C-CAP Mean* | *GEP Mean* | *t-test* | *correlation* |
| --- | --- | --- | --- | --- |
| *% Ag* | *39.4%* | *36.9%* | *0.423* | *0.880* |
| *% Forest* | *36.4%* | *22.4%* | *0.000* | *0.836* |
| *% Open* | *12.4%* | *18.7%* | *0.078* | *0.584* |
| *% Urban* | *9.9%* | *9.6%* | *0.898* | *0.832* |
| *% Wetland* | *1.2%* | *1.2%* | *0.990* | *-0.034* |
| *% Water* | *1.0%* | *0.6%* | *0.041* | *0.532* |

The percentage coverage for Agriculture calculated with the C-CAP method was plotted versus the percentage coverage for Agriculture calculated with the GEP.

The plot illustrates the difference between GIS analyses of the two datasets and the general under-representation by C-CAP (Fig E).

**Fig E. Correlation of % Agriculture in areas surrounding hive sites using C-CAP versus GEP analysis**. Linear fit: Y = 0.586x + 0.177, R^2^ = 0.775.

When glyphosate concentrations are plotted against percent acreage in agriculture using the two methods (Fig F), the general trends as expressed by exponential curves are very similar, but the GEP polygon method produces a stronger correlation (R^2^ = 0.71, AICc=-9.794).

**Fig F.** **Correlation of % Agriculture and Glyphosate concentration surrounding hive sites using C-CAP and GEP analysis**. Excel Analyse It software exponential fits produced Y = 9.648 e^0.23121x^, R^2^ = 0.48, AICc= 0.173(C-CAP, red diamonds; dash line) and Y = 11.02 e^0.1628x^, R^2^ = 0.71, AICc=-9.794(GEP Polygons, black squares; solid line).

There are many factors that would explain the differences in the land use designation and the choice of GEP polygons as the most accurate method for determining land use contemporary with honey production. These include:

- Cell size is 2.4m for C-CAP vs Digital Globe has a 30-50 cm range. A

smaller cell size allows for finer delineation and identification of objects.

- Date the image was accessed: 2010 for C-CAP but 2013-2014 for GEP with ground-truthing in areas in question.
- C-CAP would designate a ground cover as forest, but GEP showed it to be an orchard.
- C-CAP would identify open fields as "Open", but GEP showed that cattle are on it, so it is “Agriculture”.
- C-CAP does not recognize little streams or ponds but GEP resolution does.
- C-CAP sees "Forest" but Google Earth shows "Riparian"
- C-CAP see “Urban” but finer detail allows designation as “Rural/Suburban”

**Conclusion**

Although manually digitizing GEP polygon delineations is more tedious and time consuming, for the above stated reasons and the stronger correlation of the GEP derived curve, only the GEP polygon delineation method was used for final analysis of the relationship between land use and glyphosate concentration.

**Endnotes**

1. <https://catalog.data.gov/dataset/c-cap-land-cover-kauai-hawaii267aa> [↑](#endnote-ref-1)
2. <https://catalog.data.gov/dataset/c-cap-land-cover-kauai-hawaii267aa> [↑](#endnote-ref-2)
3. <https://www.gislounge.com/digitizing-errors-in-gis/> [↑](#endnote-ref-3)
4. <https://www.gislounge.com/digitizing-errors-in-gis/> [↑](#endnote-ref-4)
5. <https://www.gislounge.com/digitizing-errors-in-gis/> [↑](#endnote-ref-5)
6. <https://docs.qgis.org/2.8/en/docs/gentle_gis_introduction/vector_data.html> [↑](#endnote-ref-6)
7. <https://www.gislounge.com/digitizing-errors-in-gis/> [↑](#endnote-ref-7)
8. <http://www.esri.com/news/arcuser/0401/topo.html> [↑](#endnote-ref-8)
9. <https://en.mimi.hu/gis/planar_topology.html> [↑](#endnote-ref-9)
10. <http://www.esri.com/news/arcuser/0401/topo.html> [↑](#endnote-ref-10)
11. <http://www.esri.com/news/arcuser/0401/topo.html> [↑](#endnote-ref-11)
