# Supplementary material for: Glyphosate residue concentrations in honey attributed through geospatial analysis to proximity of large-scale agriculture and transfer off-site by bees
Source: PLoS One. 2018 Jul 11;13(7):e0198876. doi: 10.1371/journal.pone.0198876 (PMC6040695; doi:10.1371/journal.pone.0198876)

**S4: Appendix: Glyphosate data from Kauai hives and store-bought honey**

**Table A: Store-bought honey; sources and glyphosate concentration.**

| **Sample Origin** |  |  |  | **Sample #** | **Glyphosate ppb** |
| --- | --- | --- | --- | --- | --- |
| **Hawaii** | **Island:** | **Moku** | **Area** |  |  |
|  | Kauai | Kona | Waimea Valley | 5 | 15.2 |
|  |  | Kona | Koloa | 9 | 0 |
|  |  | Kona | Kalaheo | 11 | 87 |
|  |  | Kona | Poipu | 19 | 27.2 |
|  |  | Koolau | Waipake | 3 | 5 |
|  |  | Koolau | North/Northeast Kauai | 4 | 6.4 |
|  |  | Koolau | North Shore Kauai | 6 | 60.8 |
|  |  | Koolau | Kilauea | 8 | 0 |
|  |  | Koolau | Kilauea | 12 | 11.2 |
|  |  | Puna | Puhi | 1 | 15 |
|  |  | Puna | Hanamaulu | 2 | 6.2 |
|  |  | Puna | Kapa'a | 7 | 0 |
|  |  | Puna | Kapa'a | 10 | 7 |
|  |  | Puna | Puhi | 20 | 10.4 |
|  |  | Puna | Hanamaulu | 21 | 6.4 |
|  | Hawaii Island | | Hawaii Island | 15 | 12 |
|  |  |  | Kealakekua, Big Island | 16 | 7.4 |
|  |  |  | Kealakekua, Hawaii Island | 60 | 16.4 |
|  |  |  | Big Island and Oahu | 18 | 8 |
|  | Molokai |  | Molokai | 61 | 0 |
|  |  |  | Molokai | 62 | 0 |
| **Country** |  | Product of Brazil and Canada | | 17 | 0 |
|  |  | Product of Brazil and Canada | | 22 | 30.6 |
|  |  | Product of Brazil and Canada | | 14 | 8.2 |
|  |  | Product of Mexico, Brazil and Uruguay | | 13 | 0 |
|  |  | Product of USA and Argentina | | 23 | 72.4 |

**Table B: Kauai hive samples categorized by side of island and Moku with glyphosate concentration.**

| **Side of island** | **Moku** | **Sample #** | **Glyphosate ppb** | **Count** | **Median** | **Mean** | **SD** |
| --- | --- | --- | --- | --- | --- | --- | --- |
| **WINDWARD** |  |  |  |  |  |  |  |
|  | Halele'a | 2 | 0 |  |  |  |  |
|  |  | 10 | 9.2 |  |  |  |  |
|  |  | 12 | 15.2 |  |  |  |  |
|  |  | 28 | 12.6 |  |  |  |  |
|  |  | 31 | 0 |  |  |  |  |
|  |  | 32 | 0 |  |  |  |  |
|  |  | 44 | 8.2 |  |  |  |  |
|  |  | 45 | 0 |  |  |  |  |
|  |  | 51 | 0 | 9 | 0 | 5.0 | 6.3 |
|  | Ko'olau | 3 | 0 |  |  |  |  |
|  |  | 11 | 0 |  |  |  |  |
|  |  | 24 | 0 |  |  |  |  |
|  |  | 26 | 0 |  |  |  |  |
|  |  | 29 | 0 |  |  |  |  |
|  |  | 30 | 0 |  |  |  |  |
|  |  | 33 | 0 |  |  |  |  |
|  |  | 42 | 0 |  |  |  |  |
|  |  | 43 | 0 |  |  |  |  |
|  |  | 50 | 0 | 10 | 0 | 0 | 0 |
|  | Puna | 1 | 13.6 |  |  |  |  |
|  |  | 4 | 0 |  |  |  |  |
|  |  | 5 | 8.8 |  |  |  |  |
|  |  | 7 | 0 |  |  |  |  |
|  |  | 9 | 0 |  |  |  |  |
|  |  | 13 | 0 |  |  |  |  |
|  |  | 16 | 0 |  |  |  |  |
|  |  | 23 | 0 |  |  |  |  |
|  |  | 25 | 0 | 9 | 0 | 2.5 | 5.1 |

**Table B:** (continued)

| **Side of island** | **Moku** | **Sample #** | **Glyphosate ppb** | **Count** | **Median** | **Mean** | **SD** |
| --- | --- | --- | --- | --- | --- | --- | --- |
| **LEEWARD** |  |  |  |  |  |  |  |
|  | Kona | 6 | 80.2 |  |  |  |  |
|  |  | 8 | 61.4 |  |  |  |  |
|  |  | 14 | 341.6 |  |  |  |  |
|  |  | 15 | 0 |  |  |  |  |
|  |  | 17 | 0 |  |  |  |  |
|  |  | 18 | 24.6 |  |  |  |  |
|  |  | 19 | 9.6 |  |  |  |  |
|  |  | 20 | 155.2 |  |  |  |  |
|  |  | 21 | 32.6 |  |  |  |  |
|  |  | 22 | 0 |  |  |  |  |
|  |  | 27 | 0 |  |  |  |  |
|  |  | 34 | 187.2 |  |  |  |  |
|  |  | 35 | 178 |  |  |  |  |
|  |  | 36 | 171.8 |  |  |  |  |
|  |  | 37 | 92.2 |  |  |  |  |
|  |  | 38 | 77.6 |  |  |  |  |
|  |  | 39 | 0 |  |  |  |  |
|  |  | 40 | 10.4 |  |  |  |  |
|  |  | 41 | 60 |  |  |  |  |
|  |  | 46 | 13 |  |  |  |  |
|  |  | 47 | 0 |  |  |  |  |
|  |  | 49 | 0 |  |  |  |  |
|  |  | 52 | 0 |  |  |  |  |
|  |  | 53 | 0 |  |  |  |  |
|  |  | 54 | 0 |  |  |  |  |
|  |  | 55 | 0 |  |  |  |  |
|  |  | 56 | 0 |  |  |  |  |
|  |  | 57 | 27.4 |  |  |  |  |
|  |  | 58 | 0 |  |  |  |  |
|  |  | 59 | 95 | 30 | 11.7 | 53.9 | 80.9 |
|  | Mana | 48 | 292.2 | 1 | 292.2 | 292.2 | na |
|  | Napali | None | None | None |  |  |  |

**Table C: Summary statistics of glyphosate with Kauai hive samples categorized by side of island.**

| **Windward** | Count | 28 |
| --- | --- | --- |
|  | Median | 0 |
|  | Mean | 2.41 |
|  | SD | 4.87 |
| **Leeward** | Count | 31 |
|  | Median | 13 |
|  | Mean | 61.61 |
|  | SD | 90.34 |

**Table D: t-test comparing glyphosate from Windward (Eastern) and Leeward (Western) sides of Kauai.**

Data from Table B1.

| **Windward-Leeward:** |  |
| --- | --- |
| t-test probability | 0.001 |
| degrees of freedom | 57 |

**Table E: t-test comparing glyphosate between Moku pairs.**

Mana Moku had only one sample, thus could not be compared.

| Moku differences | t-test p |
| --- | --- |
| Kona -Koolau | 0.001 |
| Kona - Puna | 0.002 |
| Kona - Halelea | 0.003 |
| Koolau -Halelea | 0.043 |
| Puna -Koolau | 0.180 |
| Puna - Halelea | 0.361 |

**Table F: Kruskal-Wallis analysis of impact of side of island and Moku on glyphosate concentration.**

| Y (numerical) | X (categories) | H-stat | DF | N | p-value |
| --- | --- | --- | --- | --- | --- |
| Glyphosate | Side | 11.3 | 1 | 58 | 0.00077 |
| Glyphosate | Moku | 13.3 | 3 | 58 | 0.0041 |

**Table G: AICc analysis of fits for glyphosate concentration vs. % Agriculture.**

|  | Exp. | Power | Linear | Log | Polynomial |
| --- | --- | --- | --- | --- | --- |
| R2 | 0.594 | 0.174 | 0.417 | 0.155 | 0.429 |
| AICc | -8.664 | 7.662 | 194.88 | 195.232 | 197.055 |

**Table H: Sample #’s included within Meta-circles and their glyphosate concentrations.**

| Meta-circle # | Meta-circle Name | Sample # | Glyphosate ppb | Glyphosate ppb Mean |
| --- | --- | --- | --- | --- |
| 1 | Kilauea | 10 | 9 |  |
|  |  | 32 | 0 |  |
|  |  | 33 | 0 |  |
|  |  | 43 | 0 |  |
|  |  | 44 | 8 | 3.5 |
| 2 | Moloaa | 11 | 0 |  |
|  |  | 24 | 0 |  |
|  |  | 26 | 0 |  |
|  |  | 30 | 0 |  |
|  |  | 42 | 0 |  |
|  |  | 50 | 0 | 0.0 |
| 3 | Kapaa | 7 | 0 |  |
|  |  | 9 | 0 |  |
|  |  | 23 | 0 |  |
|  |  | 25 | 0 | 0.0 |
| 4 | Lihue | 1 | 14 |  |
|  |  | 4 | 0 |  |
|  |  | 5 | 9 |  |
|  |  | 16 | 0 | 5.6 |
| 5 | Koloa | 15 | 0 |  |
|  |  | 18 | 25 |  |
|  |  | 52 | 0 |  |
|  |  | 53 | 0 |  |
|  |  | 54 | 0 |  |
|  |  | 55 | 0 |  |
|  |  | 56 | 0 |  |
|  |  | 57 | 27 |  |
|  |  | 59 | 95 | 16.3 |
| 6 | Lawai | 27 | 0 |  |
|  |  | 39 | 0 |  |
|  |  | 40 | 10 |  |
|  |  | 46 | 13 |  |
|  |  | 49 | 0 | 4.7 |
| 7 | Agribusiness 1 | 34 | 187 |  |
|  |  | 35 | 178 |  |
|  |  | 36 | 172 | 179.0 |
| 8 | Agribusiness 2 | 8 | 61 |  |
|  |  | 14 | 342 |  |
|  |  | 20 | 155 |  |
|  |  | 21 | 33 |  |
|  |  | 37 | 92 | 136.6 |

**Table I: Samples by Side and Moku with % Agriculture, % Golf, Hiway Km, and Glyphosate concentrations.**

| Sample # | Side | Moku | Glyphosate ppb | % Agriculture | % Golf | Hiway Km |
| --- | --- | --- | --- | --- | --- | --- |
| 1 | East | Puna | 13.6 | 3.1% | 4.8% | 2.00 |
| 2 | East | Halelea | 0 | 0.0% | 0.0% | 2.39 |
| 3 | East | Koolau | 0 | 70.9% | 0.0% | 1.59 |
| 4 | East | Puna | 0 | 30.0% | 0.0% | 2.02 |
| 5 | East | Puna | 8.8 | 21.0% | 0.0% | 1.74 |
| 6 | West | Kona | 80.2 | 76.9% | 0.0% | 2.03 |
| 7 | East | Puna | 0 | 3.2% | 0.0% | 0.00 |
| 8 | West | Kona | 61.4 | 90.5% | 0.0% | 1.65 |
| 9 | East | Puna | 0 | 1.1% | 0.0% | 0.00 |
| 10 | East | Halelea | 9.2 | 4.4% | 0.0% | 2.04 |
| 11 | East | Koolau | 0 | 69.7% | 0.0% | 0.00 |
| 12 | East | Halelea | 15.2 | 19.8% | 0.0% | 0.00 |
| 13 | East | Puna | 0 | 8.6% | 0.0% | 0.00 |
| 14 | West | Kona | 341.6 | 90.5% | 0.0% | 1.65 |
| 15 | West | Kona | 0 | 43.6% | 0.0% | 4.53 |
| 16 | East | Puna | 0 | 53.8% | 0.0% | 0.00 |
| 17 | West | Kona | 0 | 0.0% | 0.0% | 0.00 |
| 18 | West | Kona | 24.6 | 0.1% | 16.2% | 4.66 |
| 19 | West | Kona | 9.6 | 2.4% | 1.6% | 3.36 |
| 20 | West | Kona | 155.2 | 90.5% | 0.0% | 1.65 |
| 21 | West | Kona | 32.6 | 90.5% | 0.0% | 1.65 |
| 22 | West | Kona | 0 | 33.9% | 0.0% | 1.44 |
| 23 | East | Puna | 0 | 0.4% | 0.0% | 0.00 |
| 24 | East | Koolau | 0 | 64.3% | 0.0% | 1.66 |
| 25 | East | Puna | 0 | 2.3% | 0.0% | 0.00 |
| 26 | East | Koolau | 0 | 68.6% | 0.0% | 1.10 |
| 27 | West | Kona | 0 | 0.0% | 0.0% | 2.29 |
| 28 | East | Halelea | 12.6 | 11.5% | 13.7% | 2.04 |
| 29 | East | Koolau | 0 | 75.0% | 0.0% | 2.03 |
| 30 | East | Koolau | 0 | 8.7% | 0.0% | 2.06 |
| 31 | East | Halelea | 0 | 0.0% | 0.0% | 2.36 |
| 32 | East | Halelea | 0 | 1.8% | 0.0% | 2.63 |
| 33 | East | Koolau | 0 | 0.0% | 0.0% | 1.98 |
| 34 | West | Kona | 187.2 | 71.9% | 1.2% | 1.08 |
| 35 | West | Kona | 178 | 71.9% | 1.2% | 1.08 |
| 36 | West | Kona | 171.8 | 71.9% | 1.2% | 1.08 |
| 37 | West | Kona | 92.2 | 90.5% | 0.0% | 1.65 |
| 38 | West | Kona | 77.6 | 61.0% | 0.0% | 2.18 |
| 39 | West | Kona | 0 | 3.5% | 0.0% | 0.32 |
| 40 | West | Kona | 10.4 | 12.9% | 0.0% | 0.52 |

**Table I:** continued

| Sample # | Side | Moku | Glyphosate ppb | % Agriculture | % Golf | Hiway Km |
| --- | --- | --- | --- | --- | --- | --- |
| 41 | West | Kona | 60 | 58.9% | 0.0% | 0.00 |
| 42 | East | Koolau | 0 | 67.9% | 0.0% | 0.00 |
| 43 | East | Koolau | 0 | 4.7% | 0.0% | 1.51 |
| 44 | East | Halelea | 8.2 | 25.3% | 0.0% | 0.00 |
| 45 | East | Halelea | 0 | 0.0% | 0.0% | 0.00 |
| 46 | West | Kona | 13 | 0.0% | 0.0% | 1.20 |
| 47 | West | Kona | 0 | 19.5% | 0.0% | 0.00 |
| 48 | West | Mana | 292.2 | 16.3% | 0.0% | 0.00 |
| 49 | West | Kona | 0 | 0.0% | 0.0% | 2.10 |
| 50 | East | Koolau | 0 | 50.4% | 0.0% | 2.26 |
| 51 | East | Halelea | 0 | 0.0% | 0.0% | 2.25 |
| 52 | West | Kona | 0 | 51.6% | 0.0% | 4.73 |
| 53 | West | Kona | 0 | 51.6% | 0.0% | 4.73 |
| 54 | West | Kona | 0 | 47.3% | 0.0% | 4.35 |
| 55 | West | Kona | 0 | 42.6% | 0.0% | 4.58 |
| 56 | West | Kona | 0 | 47.3% | 0.0% | 4.35 |
| 57 | West | Kona | 27.4 | 43.6% | 0.0% | 4.60 |
| 58 | West | Kona | 0 | 47.3% | 0.0% | 4.35 |
| 59 | West | Kona | 95 | 0.2% | 16.2% | 4.66 |

**Fig A: Multicollinearity amongst land use types.**

Samples are plotted with their % Forest vs % Agriculture. Y = 0.39 – 0.36*X, R2 = 0.23


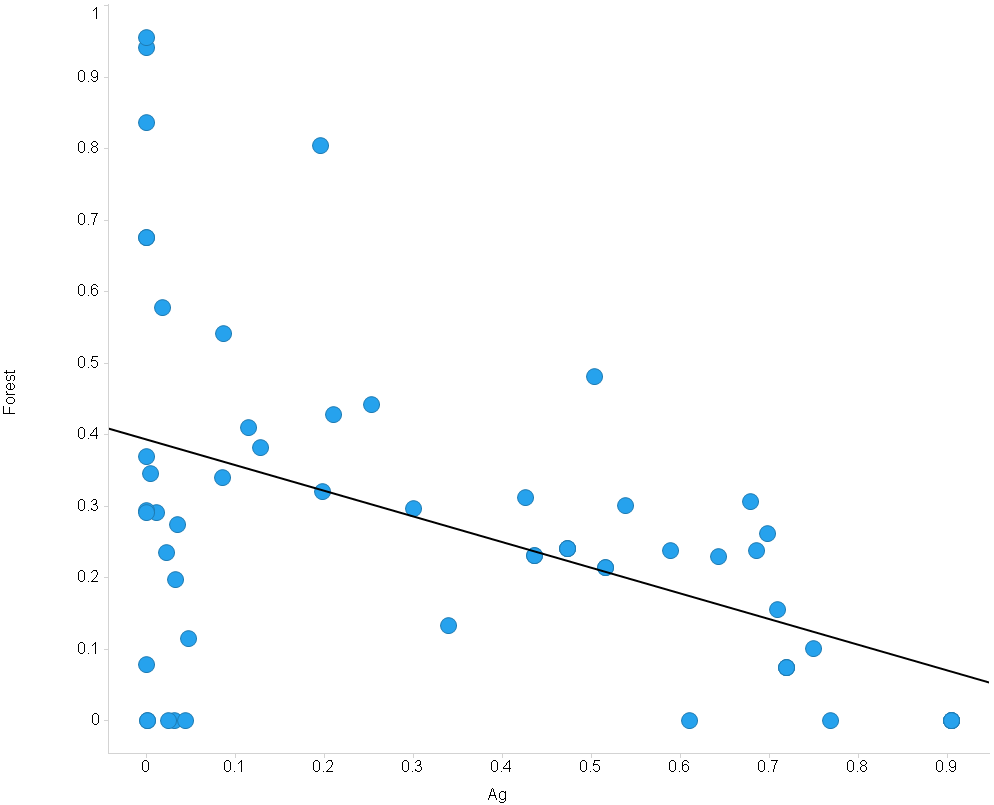

Supplement: S4 Appendix — (DOCX) [file pone.0198876.s004.docx]
